# Visualization of Positive and Negative Sense Viral RNA for Probing the Mechanism of Direct-Acting Antivirals against Hepatitis C Virus

**DOI:** 10.3390/v11111039

**Published:** 2019-11-08

**Authors:** Dandan Liu, Philip R. Tedbury, Shuiyun Lan, Andrew D. Huber, Maritza N. Puray-Chavez, Juan Ji, Eleftherios Michailidis, Mohsan Saeed, Tanyaradzwa P. Ndongwe, Leda C. Bassit, Raymond F. Schinazi, Robert Ralston, Charles M. Rice, Stefan G. Sarafianos

**Affiliations:** 1 CS Bond Life Sciences Center, University of Missouri, Columbia, MO 65201, USAJiJ@missouri.edu (J.J.); Tanya.ndongwe@outlook.com (T.P.N.); Robert.ralston@gmail.com (R.R.); 2 Department of Molecular Microbiology & Immunology, University of Missouri School of Medicine, Columbia, MO 65212, USA; 3 Center for AIDS Research, Laboratory of Biochemical Pharmacology, Department of Pediatrics, Emory University School of Medicine, Atlanta, GA 30322, USA; shuiyun.lan@emory.edu (S.L.); lbassit@emory.edu (L.C.B.); rschina@emory.edu (R.F.S.); 4 Department of Veterinary Pathobiology, University of Missouri, Columbia, MO 65211, USA; 5 Laboratory of Virology and Infectious Disease, The Rockefeller University, New York, NY 10065, USA; emichailid@mail.rockefeller.edu (E.M.); msaeed1@bu.edu (M.S.); ricec@rockefeller.edu (C.M.R.); 6 Department of Biochemistry, University of Missouri, Columbia, MO 65201, USA

**Keywords:** hepatitis C virus, Zika virus, positive-sense, negative-sense, direct acting antivirals, RNA FISH

## Abstract

RNA viruses are highly successful pathogens and are the causative agents for many important diseases. To fully understand the replication of these viruses it is necessary to address the roles of both positive-strand RNA ((+)RNA) and negative-strand RNA ((−)RNA), and their interplay with viral and host proteins. Here we used branched DNA (bDNA) fluorescence in situ hybridization (FISH) to stain both the abundant (+)RNA and the far less abundant (−)RNA in both hepatitis C virus (HCV)- and Zika virus-infected cells, and combined these analyses with visualization of viral proteins through confocal imaging. We were able to phenotypically examine HCV-infected cells in the presence of uninfected cells and revealed the effect of direct-acting antivirals on HCV (+)RNA, (−)RNA, and protein, within hours of commencing treatment. Herein, we demonstrate that bDNA FISH is a powerful tool for the study of RNA viruses that can provide insights into drug efficacy and mechanism of action.

## 1. Introduction

Positive-strand RNA, (+)RNA, viruses include many human pathogens, such as the families picornaviridae (e.g., poliovirus [1]), togaviridae (e.g., rubella virus [2]), and flaviviridae (e.g., Dengue virus [3], Zika virus [4], and hepatitis C virus [5]). While these viruses pursue a wide range of replication strategies in diverse hosts, they also share key features and are defined by their use of a (+)RNA genome. This genomic RNA fulfils three distinct functions: (1) it is the mRNA from which proteins are produced; (2) it is the template from which the negative-strand RNA ((−)RNA) is transcribed, to serve as the replicative intermediate; and (3) it codes the genetic information that must be packaged into assembling particles and transferred to target cells. At any given time, an infected cell will contain many (+)RNA molecules performing these roles, although any single (+)RNA molecule may only function in one capacity at one time. As the (+)RNA molecules are identical to one another, they cannot be differentiated by sequence and their separate roles can only be determined through simultaneous analysis of the interacting cofactors.

Following infection of a target cell, the viral RNA first serves as mRNA, exploiting the host-cell translational machinery to direct synthesis of the viral proteins; these proteins include enzymes that are responsible for synthesis of first the negative, then the positive strand of the genome, and proteins that modify the environment of the cell to support viral replication. Positive-strand RNA viruses replicate their genomes in the cytoplasm of infected cells, in association with virus-induced membrane structures, often termed the “membranous web” [6]. These membranes provide a foundation on which to anchor the viral replication complex (RC), and in combination with viral proteins, may provide protection against surveillance by the innate immune system. In the RC, the virus synthesizes new (+)RNA. At early times post-infection, the new (+)RNA will be used to generate more viral proteins and (−)RNA; at later time points, (+)RNA is packaged into particles made up of the viral structural proteins, and released from the cell. To better understand the replication of these viruses, their interactions with the host cell, and ultimately, how to combat them, it is necessary to consider both (+) and (−)RNA, and their interaction with proteins, viral and cellular.

**Figure 1:**
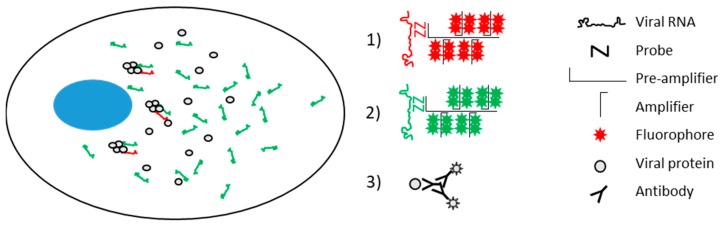
Schematic of the viral molecules in hepatitis C virus (HCV)-infected cells, and the signal amplification via RNAscope labeling of negative strand RNA (1) and positive strand RNA (2), and immunofluorescence detection of protein (3). Nucleus indicated in blue. Adapted from Wang et al. (2012) [8].

While there are thousands of antibodies available to specifically identify viral and cellular proteins, and sufficient fluorescent tags to allow co-visualization of multiple proteins in a single sample, these imaging approaches are often incompatible with conventional methods for visualization of nucleic acids (e.g., hybridization of fluorescently labeled oligonucleotide probes). Branched DNA (bDNA) in situ hybridization is a technique that exploits sequence specific probes, and branching preamplifier and amplifier DNAs, to produce an intense localized signal [7]. Unlike conventional FISH methods, bDNA FISH is readily compatible with immunofluorescence, allowing simultaneous analysis of nucleic acids and proteins (Figure 1). Various bDNA approaches have been developed for commercial use, including RNAscope [8], PrimeFlow [9], and ViewRNA [10]. These techniques have been applied to quantify and localize specific nucleic acids and the cells that harbor them. The compatibility of PrimeFlow with flow cytometry has proven particularly useful for analysis of human immunodeficiency virus (HIV)-1 latency and reservoirs [11,12,13], while RNAscope and ViewRNA have been employed diagnostically for histological staining [14,15,16,17,18,19], and in cell biology to visualize cellular and viral RNAs [20,21,22]. These techniques have frequently been employed in low resolution imaging approaches, such as histology and flow cytometry, that exploit the robust signal to clearly identify rare infected cells. A variation on the robust detection of an abundant RNA in rare cells is the detection of less abundant targets; these FISH methods have sufficient sensitivity to identify individual nucleic acid molecules, sometimes referred to as single molecule FISH. One such application of this sensitivity has been to modify the experimental conditions to achieve labelling of the viral nucleic acids in HIV-infected cells [22,23,24,25]. Most infected cells in the clinical context only contain a small number of integrated proviruses, typically just one [26]; thus, the signal amplification of bDNA FISH renders it ideally suited to the visualization of such a low abundance target.

Similar to the integrated provirus of retroviruses, the (−)RNA of (+)RNA viruses is present in low amounts relative to the (+)RNA [27,28,29,30]. It is, nevertheless, essential for the synthesis of (+)RNA and, as it lacks the multiple functions of (+)RNA, it is a more reliable marker of the RC. Here we use hepatitis C virus (HCV)-infected human hepatoma cells and Zika virus (ZIKV)-infected Vero cells to demonstrate the specific labelling of (+)RNA and (−)RNA, allowing analysis of viral activity at an individual cell level. To validate the approach, we applied bDNA FISH to a phenomenon we had previously examined [31], that of the rapid response of HCV-infected cells to a variety of anti-HCV direct-acting antiviral agents (DAAs). Using this approach we visualized the rapid decline in HCV RNA associated with the use of NS5A inhibitors [31,32,33]. This new assay represents a novel approach to evaluate other RNA viruses in future studies.

## 2. Materials and Methods

### 2.1. Cells and Viruses

Huh-7.5.1 cells are derived from the human hepatoma 7 cell line, and have been previously described [34]. Cells were propagated in Dulbecco’s modified Eagle’s medium (DMEM, Invitrogen, Carlsbad, CA, USA) supplemented with 10% fetal bovine serum (FBS). Vero-E6 cells were obtained from ATCC and cultured in DMEM supplemented with 10% FBS. Vero cells are derived from the kidney of an African green monkey and lack the genes coding type I interferon, making them suitable for the growth of many viruses [35,36,37]. Jc1-FLAG2(p7-nsGluc2A), hereafter Jc1/Gluc2A, was described previously [38]. Zika virus (isolate MR766) [39,40] was obtained from Alexander Franz (University of Missouri), and propagated and titered in Vero cells.

### 2.2. Compounds and Antibodies

Daclatasvir (DCV, BMS-790052) and Danoprevir (DNV, RG7227) were purchased from Selleckchem. Ledipasvir (LDV, GS-5885) was purchased from MedChem Express. Sofosbuvir (SOF, GS-7977) was purchased from Acme Bioscience. Mouse monoclonal primary antibody 9E10 specific for NS5A was used as previously described [41]. Mouse monoclonal antibodies specific for HCV core (C7-50) and glyceraldehyde 3-phosphate dehydrogenase (GAPDH; G-9) were purchased from Abcam and Santa Cruz Biotechnology, respectively. Alexa Fluor 647 conjugated secondary antibody and Hoechst 33258 for nuclear staining were purchased from Invitrogen.

### 2.3. Strand-Specific Quantitative RT-PCR

The initial reverse transcription step of the HCV 5′ UTR was carried out as previously described [42]. Briefly, total RNA was extracted using an RNeasy kit (Qiagen, Hilden, Germany), and quantified by absorbance at 260 nm. A quantity of 50 ng of RNA was denatured at 70 °C for 8 min with dNTPs and either the RC21 primer 5′-CTCCCGGGGCACTCGCAAGC-3′ (for the positive strand) or the tag-RC1 primer 5′-ggccgtcatggtggcgaataaGCCTAGCCATGGCGTTAGTA-3′ (for the negative strand), followed by incubation at 4 °C for 5 min. Thermoscript^TM^ reverse transcriptase (Invitrogen) was added to the denatured RNA template and incubated at 60 °C for 1 h, followed by RNase H treatment for 20 min at 37 °C. Reverse transcribed cDNA was mixed with RC1 (5′-GCCTAGCCATGGCGTTAGTA-3′) and RC21 primers for positive strand amplification and tag (5′-ggccgtcatggtggcgaataa-3′) and RC21 primers for negative strand amplification. Amplification was conducted by denaturation at 95 °C for 10 min, followed by 40 cycles of denaturation at 95 °C for 15 s and annealing/extension at 60 °C for 1 min using PerfeCTa SYBR Green FastMix (Quanta Biosciences, Beverly, MA, USA). Amplification was carried out in an Applied Biosystems^®®^ 7500 Fast Real-Time PCR Instrument (ABI). In vitro transcribed RNA from the HCV infectious clone was used to generate a standard curve.

ZIKV RT-qPCR was performed using the same general approach, with the following differences. RC21 and RC1 were replaced by Tag-ZK21 primer (5′-ggccgtcatggtggcgaataaCCTGACAACACTAAaATTGGTGC-3′) and Tag-ZK1 primer (5′-ggccgtcatggtggcgaataaAGGATCATAGGTGATGAAGAAAAGT-3′). cDNA synthesis was performed using SuperScript III First-Strand Synthesis System (Invitrogen), following the manufacturers’ instructions. qPCR amplification was conducted using PowerUp SYBR Green Master Mix (Applied Biosystems, Foster City, CA, USA), in a PikoReal 96 Real-Time PCR system (ThermoFisher Scientific). The cycle conditions were uracil-DNA glycosylase (UDG) activation at 50 °C for 2 min, dual-lock DNA polymerase at 95 °C for 2 min, followed by 40 cycles of denaturation at 95 °C for 15 sec, annealing at 55 °C for 15 sec, and extension at 72 °C for 1 min. An MR766 infectious clone [43] was used to generate a standard curve, and was subject to the same strand specific RT-qPCR protocol.

### 2.4. Branched DNA In Situ Hybridization (bDNA FISH) for Strand-Specific Nucleic Acid Visualization

**Figure 2:**
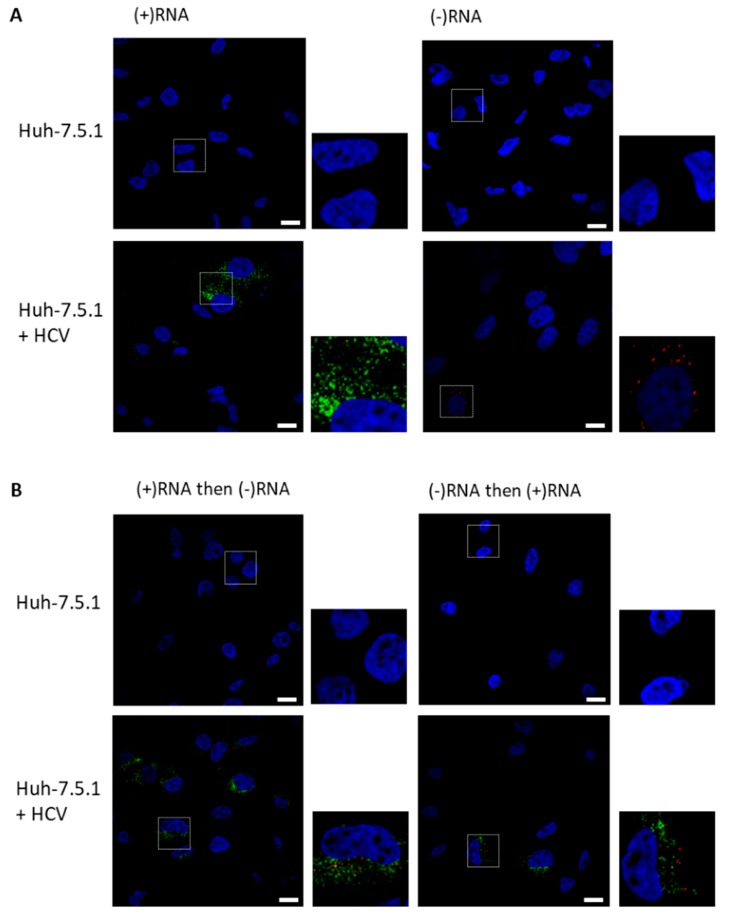
Strand-specific bDNA FISH in HCV. Huh-7.5.1 cells or Huh-7.5.1 cells infected with HCV Jc1/Gluc2A were fixed and probed for (−)RNA (red) and (+)RNA (green), and nuclei were stained with 4′,6-diamidino-2-phenylindole (DAPI, blue). Cells were imaged on a Nikon C2 confocal microscope using a 60× oil-immersion objective. (**A**) Representative images are shown for (+)RNA or (−)RNA singly stained cells. (**B**) Representative images are shown for cells stained first for (+)RNA, then for (−)RNA or cells were stained first for (−)RNA, then for (+)RNA. Boxed regions shown enlarged. Scale bars represent 10 µm.

bDNA FISH for cultured adherent cells was used for HCV RNA detection using the RNAscope method, with some modifications [8]. Cells were fixed in 4% paraformaldehyde for 30 min at room temperature (RT), washed three time in phosphate buffered saline (PBS), then incubated in PBS supplemented with 0.1% Tween-20 (PBS-T) for 10 min at RT, followed by two wash steps with PBS. Coverslips were immobilized on Superfrost glass slides using a small drop of nail polish. A circle was drawn around the coverslip using an ImmEdge hydrophobic barrier pen (Vector Laboratories). Protease treatment (Protease 3) was diluted 1:15 in PBS and incubated on the sample in a humidified HybEZ oven at 40 °C for 15 min and washed twice in PBS. Specific V-HCV-GT2a probe for (+)RNA (Catalogue number, 441361; Advanced Cell Diagnostics, Newark, CA, USA) was added to the coverslip and incubated in humidified HybEZ oven at 40 °C for 2 h, followed by HCV-GT2a-sense-C2 probe for (−)RNA (Catalogue number, 441371) diluted 1:50 in probe dilution buffer for an additional 2 h. Probes were used sequentially rather than simultaneously because they target the same region of the viral genome (Table 1) and would likely anneal to one another if applied together. Two consecutive wash steps were performed in 1× wash buffer (Catalog number, 310091; Advanced Cell Diagnostics) with agitation at RT for 2 min in every wash step after this point, and all incubations were performed in a humidified HybEZ oven at 40 °C. bDNA amplification was performed using a series of amplifiers (RNAscope; Advanced Cell Diagnostics). Amplifier hybridization 1-Fluorescent (Amp 1-FL) was added to the coverslip for 30 min, followed by Amp 2-FL hybridization for 15 min. Amp 3-FL hybridization was then added for 30 min, followed by Amp 4-FL hybridization for 15 min. If samples were to be stained by immunofluorescence, this was performed after the RNAscope staining. Anti-NS5A antibody and anti-HCV core antibody were diluted 1:1000 in PBS-T, and incubated on coverslips for 1 h at RT. Secondary anti-mouse Alexa Fluor 647 was diluted 1:2000 in PBS-T and incubated on coverslips for 1 h at RT. Nuclei were stained with DAPI for 1 min or Hoechst 33258 at 0.5 µg/mL for 10 min at room temperature in PBS-T. Coverslips were washed 3 times in PBS-T after each incubation. Finally, coverslips were detached and mounted on fresh slides using ProLong Gold Antifade reagent (Thermo Fisher Scientific, Waltham, MA, USA). Most images were obtained using a Leica TCP SP8 MP confocal fluorescence equipped with a 63× HC PL APO CS2 oil-immersion objective (numerical aperture 1.4), and a tunable supercontinuum white light laser. The excitation/emission bandpass wavelengths used to detect DAPI, Alexa 488, ATTO 550, and Alexa 647 were set to 405/420–480, 488/505–550, 550/560–610, 568/580–630, and 647/655–705 nm, respectively. Pixel size under these conditions was 0.18 μm. Within any given dataset, conditions such as the laser intensity, exposure time and pinhole were kept constant, to allow comparison of the images. Images of the HCV-infected cells for Figure 2 were captured using a Nikon C2 confocal microscope, with a 60× APO oil-immersion objective (numerical aperture 1.4). Excitation lasers were 405 nm, 488 nm, and 561 nm.

ZIKV-infected cells were stained by the same protocol as HCV, with the following changes: the protease pre-treatment used a 1:2 dilution, rather than 1:15; and the ZIKV-specific probes were V-ZIKA-pp-O2 for the (+)RNA (Catalogue number, 464531) and V-ZIKA-pp-O2-sense-C2 for the (−)RNA (Catalogue number, 478731-C2).

In order to quantify the differential drug effects on (+) and (−) strands of HCV RNA, we manually acquired 30 images of each biological replicate drug treatment experiment and performed cellular analysis and image analysis using BioTek Gen5 software. Abundance of (+) and (−)RNA was quantified and plotted in two ways: number of fluorescent foci per infected cell, and fluorescence intensity per infected cell. ZIKV staining was performed similarly, with an additional calculation of the area of (−)RNA foci.

### 2.5. Statistical Analyses

Graphs were plotted using Microsoft Excel. For significance testing, GraphPad Prism 6 was used to perform one-way ANOVA with Dunnett’s post-test for multiple comparisons, comparing each test condition to a control sample, or Tukey’s post-test for multiple comparisons comparing all conditions to one another.

## 3. Results

### 3.1. RNAscope Permits Simultaneous Imaging of (+) and (−)RNA Strands

As proof of concept for the labelling of (+)RNA and (−)RNA, we chose to use HCV-infected cells, as HCV is an important human pathogen and one of the most extensively studied (+)RNA viruses. Furthermore, in their initial description of the RNAscope method, Wang and colleagues showed the labeling of the HCV (+)RNA, co-labeled with a probe against 18S rRNA [8]. Labeling the less abundant (−)RNA illustrates the sensitivity of the technique; additionally, (−)RNA is likely a more faithful marker of RCs than the (+)RNA, as the (−)RNA is thought to serve only one function in the cell, that of replicative intermediate from which (+)RNA can be synthesized. We fixed infected cells, then permeabilized and treated with protease to remove some of the proteins that might otherwise obstruct probe binding in the RC. There was little discernable staining of HCV negative Huh-7.5.1 cells with either (+)RNA or the (−)RNA probe sets (Figure 2A). By contrast, staining of both strands was apparent when HCV-infected cells were stained for either (+)RNA or (−)RNA. We then probed for both strands simultaneously, either (+)RNA then (−)RNA, or (−)RNA then (+)RNA (Figure 2B). We observed non-overlapping staining in both cases; staining intensity of (+)RNA and (−)RNA appeared similar in both protocols.

**Figure 3:**
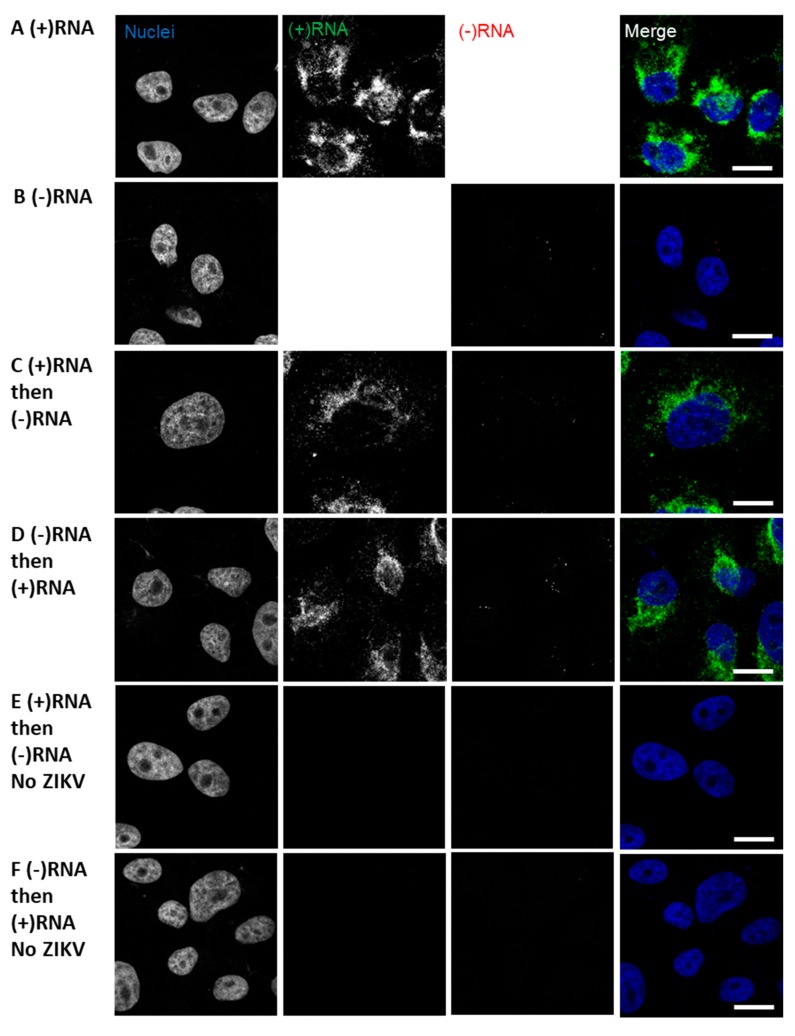

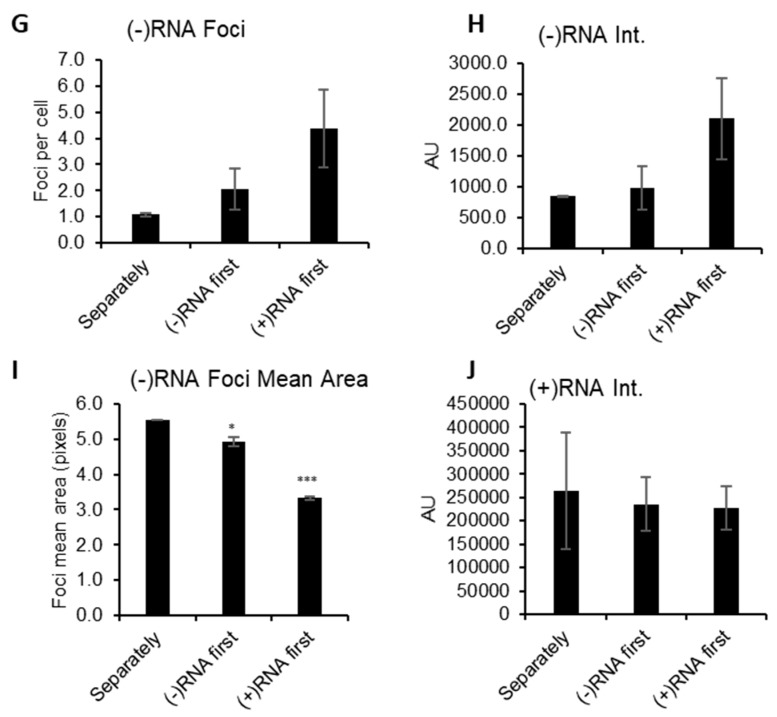
Strand-specific bDNA FISH in ZIKV. Vero cells were infected with ZIKV at a multiplicity of infection (MOI) of 0.1. After 48 h, the cells were fixed and probed for (−)RNA (red) and (+)RNA (green). Finally, nuclei were stained with DAPI. Cells were imaged on a Leica SP8 confocal microscope using a 63× oil-immersion objective. Representative images are shown for sequential staining approaches: (**A**) (+)RNA only; (**B**) (−)RNA only; (**C**) cells were stained first for (+)RNA, then for (−)RNA; (**D**) cells were stained first for (−)RNA, then for (+)RNA. (**E**) and (**F**) Uninfected cells were stained for (+)RNA and (−)RNA as indicated. For 100–250 cells per condition, (**G**) (−)RNA foci were counted, (**H**) (−)RNA fluorescence intensity was measured, (**I**) area of (−)RNA foci was measured, and (**J**) (+)RNA fluorescence intensity was measured. Error bars represent standard error of the mean for two independent experiments. Significances of differences between staining methods were calculated using one-way ANOVA and Dunnett’s post-test for multiple comparisons. Differences were not significant unless otherwise indicated. *, *p* ≤ 0.05; ***, *p* ≤ 0.001. AU, arbitrary units; px, pixels. Scale bars represent 10 µm.

In the course of developing the sequential RNA staining protocol we performed similar staining with ZIKV-infected cells (Figure 3). We were able to readily stain Zika virus (+)RNA using the same conditions as HCV, however, to stain ZIKV (−)RNA it was necessary to increase the concentration of protease used to pre-treat samples from the recommended 1:15 dilution to 1:2. We also found that the staining of the abundant (+)RNA was consistent independent of single or co-staining, and the order of staining (Figure 3A,C,D); however, the (−)RNA staining was altered depending on whether the (−)RNA was stained first or second. Comparing the co-staining shown in Figure 3C,D, (−)RNA staining appears to more closely resemble the single staining (−)RNA (Figure 3B) when (−)RNA is stained before (+)RNA. Staining of uninfected cells demonstrated a high level of specificity, as no foci were seen (Figure 3E,F). We further examined the conclusion that it is necessary to stain the (−)RNA before the (+)RNA in order to get reliable labeling of (−)RNA in a co-staining experiment, by quantifying various parameters of the images and foci. (−)RNA foci were quantified by number, fluorescence intensity, and size in the co-stained samples and compared to the singly stained (−)RNA samples (Figure 3G–I). These comparisons again suggested that staining the (−)RNA first in co-staining yields results that are more consistent with the singly stained samples. When (+)RNA was stained first, there were more numerous foci and greater intensity, but the foci were smaller. We suspect that the (−)RNA probes may have been annealing to residual (+)RNA probe. In contrast, the intensity of (+)RNA staining did not vary depending of the sequence of staining (Figure 3J). Finally, we performed strand-specific RT-qPCR on the ZIKV RNA to determine the relative abundance of (+)RNA and (−)RNA: we found (+)RNA was 33-fold more abundant than (−)RNA (Table 2).

### 3.2. Staining of Nucleic Acids and Protein in HCV-Infected Cells

**Figure 4:**
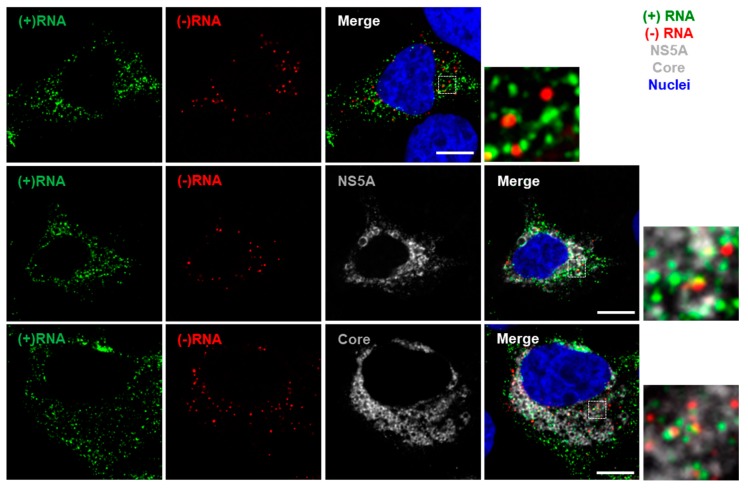
Multiplex imaging of both (+) and (−)RNA strands and protein in HCV-infected cells. Huh-7.5.1 cells were infected with HCV Jc1/Gluc2A at a multiplicity of infection (MOI) of 0.5. After 48 h, cells were fixed and probed sequentially for (−)RNA (red), (+)RNA (green) and viral protein (white). Finally, nuclei were stained with DAPI (blue). Cells were imaged on a Leica SP8 confocal microscope using a 63× oil-immersion objective. Boxed regions shown enlarged. Scale bars represent 10 µm.

Following establishment of protocols for sequential staining of (−)RNA and (+)RNA, in virus harboring cells, we additionally stained protein for simultaneous imaging. HCV-infected Huh-7.5.1 cells were stained for RNA and NS5A or core proteins (Figure 4). During the FISH staining, the proteolytic step was optimized by varying the amount of protease needed to remove sufficient protein to expose the RNA for labeling, while retaining sufficient protein for immunofluorescence labeling in subsequent steps. In principle, this could allow visualization of HCV particles, as the signal amplification in RNAscope permits single genome imaging. This may have applications for studying virus entry; however, in the context of the high intracellular levels of (+)RNA and core in these infections, it is impossible to confidently isolate the particle-associated signal.

### 3.3. Analysis of Differential Phenotypes of HCV Inhibitors

**Figure 5:**
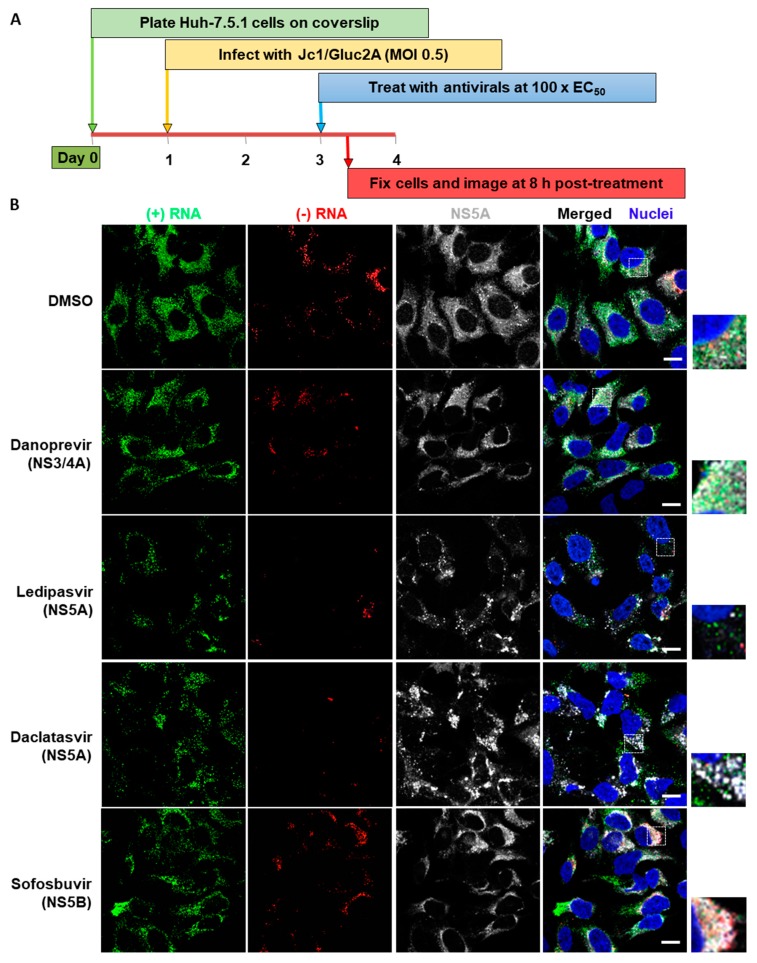

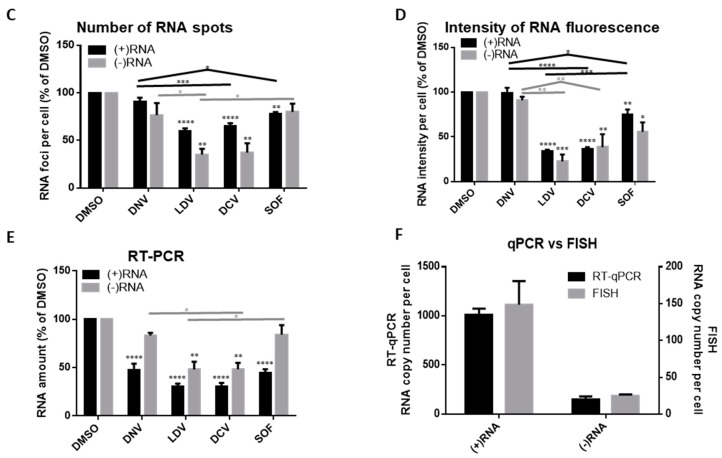
Early impact of different classes of HCV inhibitors on HCV RNA and NS5A. (**A**) Huh-7.5.1 cells were infected with HCV Jc1/Gluc2A at an MOI of 0.5. After 48 h, the cells were treated with various inhibitors (Danoprevir, 0.32 μM; Ledipasvir, 3 μM; Daclatasvir, 3.2 nM; Sofosbuvir, 20 μM), then after a further 8 h cells were fixed and probed sequentially for (−)RNA, (+)RNA and NS5A. Finally, nuclei were stained with DAPI. Cells were imaged on a Leica SP8 confocal microscope using a 63× oil-immersion objective. (**B**) Representative images from each inhibitor showing (−)RNA in green, (+)RNA in red, NS5A in gray and nuclei in blue. Boxed regions shown enlarged. Scale bars represent 10 µm. Early impact of different classes of HCV inhibitors on HCV RNA and NS5A. (C and D) Fields of view were captured as described in Figure 3, then analyzed using Gen5 software (BioTek). Abundance of (+) and (−)RNA strands was quantified by: (**C**) number of RNA foci per cell; (**D**) RNA fluorescence intensity per cell in the field of view; and (**E**) RT-qPCR to determine copy numbers of the of (+) and (−)RNA using specific primers. All values are expressed as a percentage of the DMSO-treated sample. Error bars indicate standard error of the mean for three independent experiments. Significance of differences between DMSO and drug treatments was calculated using one-way ANOVA and Tukey’s post-test for multiple comparisons. Differences were not significant unless otherwise indicated. *, *p* ≤ 0.05; **, *p* ≤ 0.01; ***, *p* ≤ 0.001; ****, *p* ≤ 0.0001. *P* values shown on columns indicate comparison to DMSO. Lines indicate comparisons between test samples. Lines group samples, and are color coordinated for (+)RNA and (−)RNA. Flat lines group all samples beneath them, inverted Vs indicate comparison of the samples under the ends of the lines. DNV, Danoprevir; LDV, Ledipasvir; DCV, Daclatasvir; SOF, Sofosbuvir. (**F**) Comparison of intracellular HCV transcript number under control (DMSO) conditions as determined by strand-specific RT-qPCR and bDNA FISH. Error bars indicate standard error of the mean; no additional statistical analyses were performed.

The sensitivity of these imaging methods permits the study of early events following drug addition. Cells were infected with HCV, then after 48 h, were treated with DAAs targeting specific viral proteins, at concentrations 100 times higher than the concentration required to inhibit HCV infection by 50% (EC_50_). Cells were fixed and examined 8 h after the addition of inhibitors (Figure 5A); this allowed us to examine the effects of the DAAs on cells with established HCV infection, rather than simply the loss of virus in newly infected cells. Distinct mechanisms of action were revealed by the effects on both RNA and protein staining (Figure 5B). Following treatment with NS5A inhibitors, NS5A staining becomes more concentrated in large aggregations, compared to the more diffuse staining apparent in the control sample or following treatment with NS3 and NS5B inhibitors. In addition to the change in NS5A distribution, it appeared that NS5A inhibitors provoked a general reduction in the amounts of both (+)RNA and (−)RNA, relative to control or treatment with other inhibitors. These data are consistent with previous reports demonstrating the fast-acting phenotype of NS5A inhibitors [31,44].

Changes in RNA levels were quantified by several different approaches. Fields of view were captured, then analyzed automatically using Gen5 Image+ Software (BioTek). Two types of analyses were performed, quantifying either the number of fluorescent foci per cell, or the fluorescence intensity per cell, for both RNA strands (Figure 5C,D). These analyses supported the initial observation, that even at this early time post-treatment, the NS5A inhibitors induced a reduction in the levels of RNA, to a greater degree than the NS3 and NS5B inhibitors. The potency of NS5A inhibitors in suppressing viral RNA levels was also supported by real-time PCR quantitation (RT-qPCR) (Figure 5E); although RT-qPCR indicated that all of the inhibitors suppressed (+)RNA, the NS5A inhibitors showed the strongest suppression. Compared to other classes of DAA, the NS5A inhibitors appeared particularly potent at suppressing (−)RNA levels (Figure 5), as all three analyses showed a pronounced reduction in the (−)RNA with LDV and DCV, that was absent for DNV and SOF at this time point. These data demonstrate the feasibility of analyzing effects of drugs on strand-specific RNA detection and replication of RNA viruses at the level of individual cells and single viral RNA molecules. It also enables differentiation between inhibition of new infections, and effects on cells with on-going viral replication. Finally, we compared the absolute RNA copy numbers determined for (+)RNA and (−)RNA by RT-qPCR and bDNA FISH, using the DMSO control data in each case (Figure 5F). The absolute RNA values were of a comparable order of magnitude to those reported elsewhere [45,46], although the exact numbers naturally depend heavily on the isolate of HCV and the cell type used, and the efficiency of the assay method(s). The genome copy numbers determined using RT-qPCR and FISH differed by approximately five-fold, possibly because the RT-qPCR harvests total RNA while the microscopy approach counts only the RNA in a single plane, not the complete volume of the cell; however the ratio of (+)RNA to (−)RNA was the same for the two methods, suggesting that this ratio is reliable.

## 4. Discussion

Studying the (−)RNA of (+)RNA viruses is particularly challenging, owing to its relatively low abundance in infected cells [27,28,29], and the low sensitivity of traditional fluorescent in situ hybridization imaging techniques when applied to low abundance transcripts [47]. Nevertheless, understanding the behavior of (−)RNA is of critical importance to elucidating the HCV replication mechanism, as it is a most critical component of the RC due to its function as the template for the production of new (+)RNA. Consequently, disruption of the RC may best be understood by study of the (−)RNA. The low amount of (−)RNA in cells might reflect the limited role it plays in the viral replication cycle and/or may be a viral strategy to avoid forming dsRNA, a potential inducer of innate immunity [48,49,50].

In the current study, we specifically labeled the (−)RNA of HCV, both in singly and dual labeled infected cells. In addition, we performed cell-level quantitation of the two RNA species, and compare this imaging-based quantitation to RT-qPCR; we found an approximately five-fold excess of the HCV (+)RNA by both techniques, consistent with a previous report that used RT-qPCR [27].

In contrast with HCV, ZIKV (+)RNA was so abundant in infected cells that it was not possible to count foci, making direct comparisons to the abundance of (−)RNA unreliable. We also found that the ZIKV (−)RNA required more rigorous pre-treatment with protease than HCV (−)RNA to permit FISH staining. We assume this reflects differences in the nature of the RC, the amount of protein bound, and accessibility to protease treatment; these properties very likely vary extensively between viruses. Nevertheless, both bDNA FISH and strand-specific RT-qPCR approaches revealed a large excess of (+)RNA over (−)RNA in Zika virus-infected cells, comparable to a previous estimate using another flavivirus, the West Nile virus [51]. Collectively, the data obtained from HCV and ZIKV studies suggest that RT-qPCR and bDNA FISH are both suitable to follow transcript abundance, however, care must be taken when comparing transcript numbers, as efficiency of amplification and labelling can vary significantly between viruses and methods.

It was striking that our ability to detect colocalized RNA strands was extremely limited. In the case of ZIKV we saw very few (−)RNA strands by bDNA FISH, while in HCV harboring cells, (−)RNA was reasonably abundant, but rarely colocalized with the (+)RNA signal. As the replication of the viruses requires transcription of one strand from the other, we would anticipate a certain proportion of the (+)RNA and (−)RNA signals should colocalize. While we have no single definitive explanation for the lack of colocalized RNA molecules, there are potential explanations. In the Zika imaging, we found that it was necessary to increase the concentration of protease. This may indicate that the (−)RNA, in particular, is sequestered and unavailable for labeling, potentially due to extensive interactions with the RC. A related potential contributing factor is the extremely high melting temperature reported for long double-stranded RNA [52,53,54]. This could lead to a situation where the dsRNA is generally refractory to FISH labelling (which requires a single-stranded RNA molecule), reducing the incidence of apparent colocalization, and reducing the apparent abundance of (−)RNA, a greater proportion of which is likely to be double-stranded in replication complexes. This interpretation would suggest that the (−)RNA molecules seen are predominantly “free” single-stranded RNAs; the abundance of this species may vary greatly between viruses and cell types. Efficient labeling of dsRNA species, such as may be found in the RC, may require the use of aggressive denaturation to release single-stranded RNAs for FISH.

An area of particular importance in antiviral research is the mechanism of action of DAAs, understanding their impact on virus replication and their role in therapeutic regimens. A strength of this imaging-based technique is to permit analysis of viral RNA and protein in the same cell, and to select those cells desired for analysis. All DAAs will prevent the spread of infection over time, irrespective of their specific mechanism, so long as they inhibit some part of the replication cycle. In contrast to other methods, such as RT-qPCR or Western/Northern blot analyses, which can phenotypically characterize only mixtures of cell populations, the use of an imaging-based approach allows us to specifically examine cells that are already infected, and determine how different DAAs influence on-going viral replication. Recently, we used another imaging-based strategy to assess the effects of DAAs against HCV by following NS5A staining [31]. Similar to others [44], we were able to show that the NS5A-targeting drugs have the fastest effect on suppression of viral proteins and total viral RNA and on redistribution of NS5A. Here, we wished to determine whether staining for viral RNA could provide additional insights. To this end, we followed the effects of antivirals targeting the protease, polymerase and NS5A on viral (+) and (−)RNA. We confirmed that the NS5A inhibitors are the most potent and fast acting inhibitors, both by counting RNA foci per cell and RT-qPCR. Whereas the extent of (−)RNA suppression was found to be similar with both methods (Figure 5C,E, gray bars), reductions in the (+)RNA appeared less pronounced when RNA foci per cell were counted. This was likely due to underestimation of the foci number when a large number of (+)RNA was present (such as in the DMSO-treated control), leading to underestimating the effect of the inhibitors. As such, the focus counting approach may be more suited to viruses or conditions where less RNA is present.

As a solution to the difficulties in counting discrete RNA foci, we used an alternative form of quantification, measuring the total fluorescence in the field of view, expressed per cell. This greatly improved the consistency of the data, and confirmed that the NS5A inhibitors were particularly effective at rapidly inhibiting HCV, as we have previously reported [31]. The lack of suppression by the NS3 inhibitor Danoprevir is not a surprise in this context, given that inhibiting the viral protease will have a limited impact on the function of extant RCs in the short time frame (8 h) of these experiments [55,56]. It is noteworthy that the NS5A inhibitors also suppress viral RNA to a greater extent than Sofosbuvir, a potent inhibitor of the HCV polymerase, NS5B [57]. As the concentration of Sofosbuvir used should completely inhibit polymerase function, the apparently greater potency of the NS5A inhibitors as rapid suppressors of the viral RNA is consistent with them functioning through a distinct or additional mechanism that affects the quantity of viral RNA [32,33,58]; it is possible that these inhibitors destabilize the RC and hasten the degradation of the viral RNA, particularly the (−)RNA that is normally protected from nucleases by the RC [59]. This hypothesis is consistent with the clear disruption of NS5A localization following treatment with the NS5A inhibitors seen in this study and previously by us and others [31,60].

It has been previously reported that NS5A inhibitors suppress levels of (−)RNA to a greater degree than the levels of (+)RNA [61]. In our assays we did not observe significant differences in the impact of NS5A inhibitors on (+) versus (-)RNA. The discordance in the reported results may come from the disparate timing of the two experiments. The study of Ramanan and colleagues added the inhibitors 20 h post infection [61]; we allowed infection to proceed for 48 h before the addition of inhibitors, as we were interested in the effects of these inhibitors in the context of an established infection. It is possible that the sensitivity of (−)RNA to NS5A inhibitors is more pronounced before the infection is fully established.

Collectively our data demonstrate the utility of branched DNA in situ hybridization as a tool to help address questions relating to the biology of RNA viruses. The high sensitivity of bDNA FISH allows sensitive visualization of incoming viral RNA, before any amplification has taken place, and early stages of viral genome replication. The ability to select specific cell populations for analysis enables in-depth investigation of viral RNA strand-specific effects, localization, and interactions with proteins, owing to the compatibility with immunofluorescence. Thus, this approach may be particularly suitable, not only to the study of drug mechanisms of action, but also to studies involving over-expression or silencing of host cell-factors. The high specificity and sensitivity of staining offered by these methods should help illuminate the critical role of (−)RNA, until now a concealed player in the biology of (+)RNA viruses.

## Figures and Tables

**Table 1:** Probes used in branched DNA fluorescence in situ hybridization (bDNA FISH). All probes were purchased from ACDBio. HCV, hepatitis C virus; ZIKV, Zika virus.

Nucleic Acid Target	Probe Name	Catalog#	Target Region of Genome (Coded Protein)
HCV (+)RNA	V-HCV-GT2a	441361	4268–5505 (NS3-NS4B)
HCV (−)RNA	HCV-GT2a-sense-C2	441371	4268–5505 (NS3-NS4B)
ZIKV (+)RNA	V-ZIKA-pp-O2	464531	866–1763 (M-E)
ZIKV (−)RNA	V-ZIKA-pp-O2-sense-C2	478731-C2	866–1763 (M-E)

**Table 2:** Copies per cell of ZIKV RNA, estimated by strand specific RT-qPCR from three independent experiments. S.E.M., standard error of the mean.

	Copies per Cell	S.E.M.	Fold Difference	S.E.M.
**(+)RNA**	3.0 × 10^6^	5.7 × 10^5^	33	2.4
**(−)RNA**	9.0 × 10^4^	2.2 × 10^4^		
